# Widespread signatures of positive selection in common risk alleles associated to autism spectrum disorder

**DOI:** 10.1371/journal.pgen.1006618

**Published:** 2017-02-10

**Authors:** Renato Polimanti, Joel Gelernter

**Affiliations:** 1 Department of Psychiatry, Yale School of Medicine, West Haven, Connecticut, United States of America; 2 VA CT Healthcare Center, West Haven, Connecticut, United States of America; 3 Departments of Genetics, Yale School of Medicine, New Haven, Connecticut, United States of America; 4 Department of Neuroscience, Yale University School of Medicine, New Haven, Connecticut, United States of America; Case Western Reserve University School of Medicine, UNITED STATES

## Abstract

The human brain is the outcome of innumerable evolutionary processes; the systems genetics of psychiatric disorders could bear their signatures. On this basis, we analyzed five psychiatric disorders, attention deficit hyperactivity disorder, autism spectrum disorder (ASD), bipolar disorder, major depressive disorder, and schizophrenia (SCZ), using GWAS summary statistics from the Psychiatric Genomics Consortium. Machine learning-derived scores were used to investigate two natural-selection scenarios: complete selection (loci where a selected allele reached fixation) and incomplete selection (loci where a selected allele has not yet reached fixation). ASD GWAS results positively correlated with incomplete-selection (p = 3.53*10^−4^). Variants with ASD GWAS p<0.1 were shown to have a 19%-increased probability to be in the top-5% for incomplete-selection score (OR = 1.19, 95%CI = 1.11–1.8, p = 9.56*10^−7^). Investigating the effect directions of minor alleles, we observed an enrichment for positive associations in SNPs with ASD GWAS p<0.1 and top-5% incomplete-selection score (permutation p<10^−4^). Considering the set of these ASD-positive-associated variants, we observed gene-expression enrichments for brain and pituitary tissues (p = 2.3*10^−5^ and p = 3*10^−5^, respectively) and 53 gene ontology (GO) enrichments, such as nervous system development (GO:0007399, p = 7.57*10^−12^), synapse organization (GO:0050808, p = 8.29*10^−7^), and axon guidance (GO:0007411, p = 1.81*10^−7^). Previous genetic studies demonstrated that ASD positively correlates with childhood intelligence, college completion, and years of schooling. Accordingly, we hypothesize that certain ASD risk alleles were under positive selection during human evolution due to their involvement in neurogenesis and cognitive ability.

## Author summary

Predisposition to psychiatric disorders is due to the contribution of many genes involved in numerous molecular mechanisms. Since brain evolution has played a pivotal role in determining the success of the human species, the molecular pathways involved with the onset of mental illnesses are likely to be informative as we seek an understanding of the mechanisms involved in the evolution of human brain. Accordingly, we tested whether the genetics of psychiatric disorders is enriched for signatures of positive selection. We observed a strong finding related to the genetics of autism spectrum disorders (ASD): common risk alleles are enriched for genomic signatures of incomplete selection (loci where a selected allele has not yet reached fixation). The genes where these alleles map tend to be expressed in brain and pituitary tissues, to be involved in molecular mechanisms related to nervous system development, and surprisingly, to be associated with increased cognitive ability. Previous studies identified signatures of purifying selection in genes affected by ASD rare alleles. Accordingly, at least two different evolutionary mechanisms appear to be present in relation to ASD genetics: 1) rare disruptive alleles eliminated by purifying selection; 2) common alleles selected for their beneficial effects on cognitive skills. This scenario would explain ASD prevalence, which is higher than that expected for a trait under purifying selection, as the evolutionary cost of polygenic adaptation related to cognitive ability.

## Introduction

The human brain is a uniquely complex organ; it is the outcome of numerous evolutionary processes that were necessary for the success of the human species [1]. The same mechanisms that contributed to the evolution of human brain are likely to be involved in the pathogenesis of mental illnesses [2, 3]. Numerous evolutionary hypotheses have been proposed to account for the observation that phenotypic traits with deleterious effects on fitness such as psychotic and mood disorders, have not been removed by natural selection [4]. Risk alleles with large effects on predisposition to mental illness should be under negative selection, at least insofar as they interfere with reproductive fitness. This does appear to be true for rare and *de novo* mutations strongly associated with psychiatric disorders [5], but most genetic risk for these disorders is attributable to a polygenic predisposition [6]. Specifically, heritability analyses have shown that major psychiatric diseases are highly polygenic: their genetic predisposition should be due the additive result of hundred to thousand variants with small effect size [7–9].

Similarly, genome evolution also seems to operate mainly on gene networks rather than single genes [10]. Different authors have hypothesized that human adaptation in response to the selection of polygenic phenotypes may occur via subtle allele frequency shifts at many loci [11–13]. Signatures of polygenic adaptation have been identified in the context of several phenotypic traits, including immune response [14, 15], anthropometric traits [16–19], metabolic traits [15, 17], skin pigmentation [17], telomere length [20], bone mineral density [21], and dietary patterns [22]. Two previous studies investigated the genome-wide enrichment of evolutionary signatures among schizophrenia (SCZ) risk alleles using the results of the large genome-wide association study (GWAS) meta-analysis conducted by the Psychiatric Genomics Consortium (PGC) [23]. In 2015, Xu and colleagues reported that genes near human accelerated regions conserved in non-human primates (pHARs) are enriched for SCZ-associated loci, and they are particularly related to the GABA (gamma-Aminobutyric acid)-related co-expression module [24]. In 2016, Srinivasan and colleagues obtained consistent results using a different approach based on Neanderthal selective sweep (NSS) score, which is an indicator of positive selection in early humans based on the depletion of Neanderthal-derived alleles [25]. They observed loci associated with schizophrenia are more prevalent in regions with a low NSS score (i.e., this is evidence of positive selection) [25].

In the present study, we investigated polygenic adaptation signatures in the systems genetics of five psychiatric disorders: attention deficit hyperactivity disorder (ADHD), autism spectrum disorder (ASD), bipolar disorder (BP), major depressive disorder (MDD), and SCZ.

## Results

Our investigation was conducted using summary statistics from GWAS of psychiatric disorders and testing for enrichment of positive selection signatures, using the same concept as is applied in high-resolution polygenic risk score analysis [26, 27]. The positive selection signatures were identified using the hierarchical boosting (HB) algorithm, which is a machine-learning classification framework that combines the functionality of several selection tests to uncover different genetic features that are expected under selective sweeps [28].

**Fig 1:**
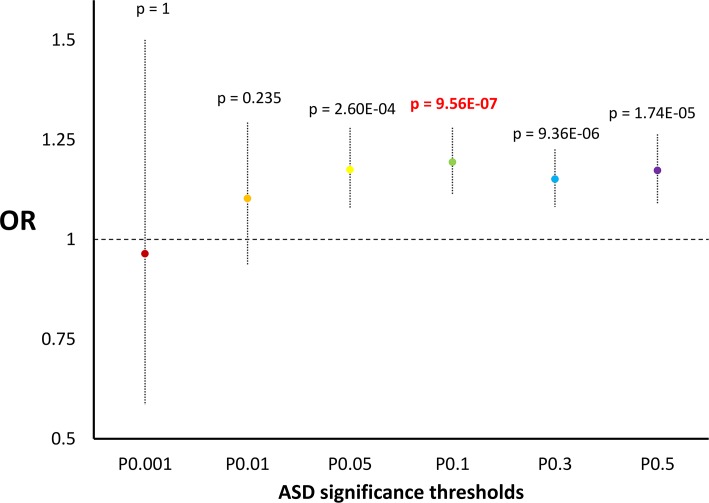
Enrichments for scores related to incomplete selection in ASD GWAS considering different thresholds. Odds Ratio, 95% confidence interval, and p value is reported for each threshold investigated.

**Fig 2:**
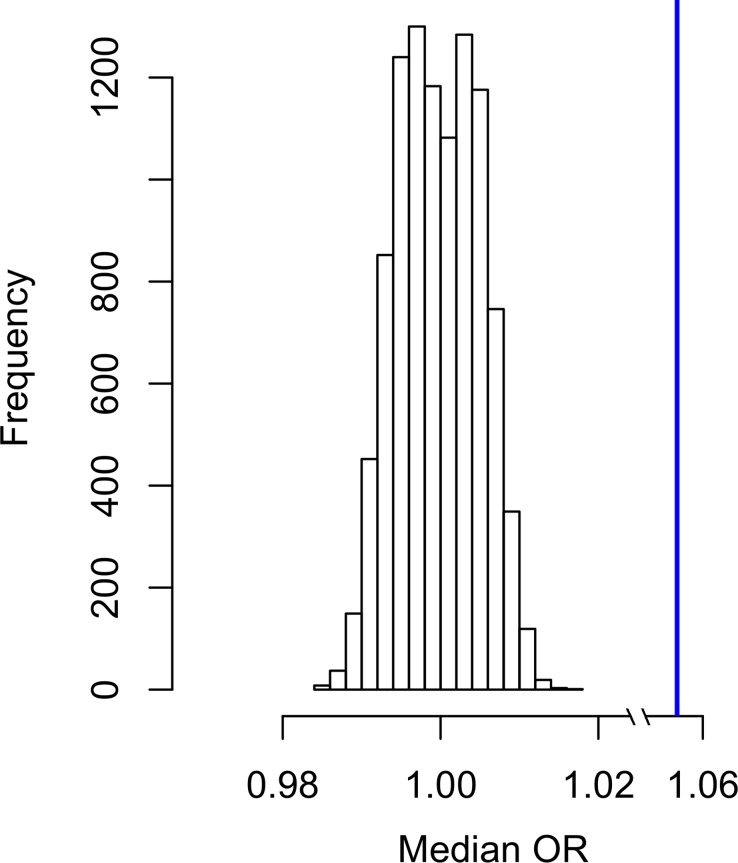
Null distribution of median OR (considering minor allele effect) generated from 10,000 permutations of ASD dataset. Blue line represents the median OR observed in the actual ASD dataset.

**Fig 3:**
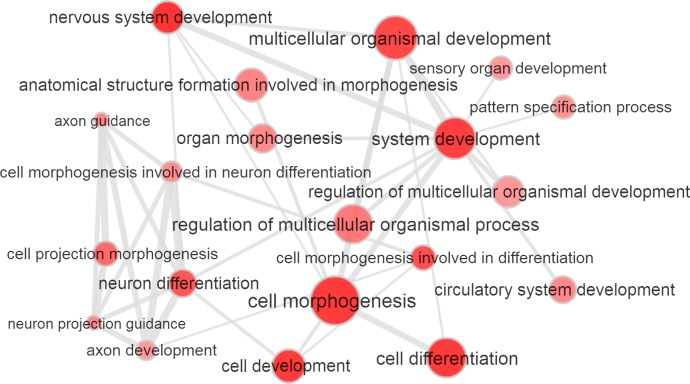
Similarity network based on GO enrichment results. Bubble color indicates the GO p-value; bubble size indicates the frequency of the GO term in the Gene Ontology Annotation (UniProt-GOA) Database which annotates all UniProt entries with GO terms. Highly similar GO terms are linked by edges in the graph, where the line width indicates the degree of similarity.

Initially, we verified whether the GWAS significance (-log_10_ p value) reported for the variants investigated correlate with the HB scores related to the corresponding genomic regions for incomplete and complete selection (loci where a selected allele *has not* yet reached fixation and loci where a selected allele *has* reached fixation, respectively) using a non-parametric test (Spearman's correlation). Significant positive correlations were observed between ASD GWAS results and HB scores for incomplete selection (p = 3.53*10^−4^): higher GWAS significance correlated with higher HB scores (S1 Table). We confirmed these results by conducting 10,000 random permutations of the GWAS results with respect to the corresponding HB scores and testing whether the observed correlations were significantly higher from the ones in the null distribution of the permuted datasets (ASD vs. incomplete selection–permutation p = 1*10^−4^, S1 Fig). Then, we investigated which GWAS significance threshold is more enriched for natural selection signatures. We considered the top-5% of HB scores as suggestive evidence of natural selection. Variants with ASD GWAS p < 0.1 have a 19%-increased probability to be in the top-5% of the HB scores for incomplete selection (Odds Ratio (OR) = 1.19, 95%CI = 1.11–1.8, p = 9.56*10^−7^; Fig 1; S2 Table). To evaluate the direction of these natural-selection enrichments, we tested whether the variants included within the significant thresholds (ASD GWAS p < 0.1) and with suggestive evidence of positive selection (top-5% HB scores for incomplete and complete selection, respectively) show an overrepresentation with respect to a specific effect direction (positive association [GWAS OR > 1] *vs*. negative association [GWAS OR < 1). Taking into account the effects of minor alleles, we compared the median OR of the variants identified with respect to the median value of ORs calculated on the basis of 10,000 permutations of the original ASD. The median value of OR for variants with ASD GWAS p < 0.1 and HB score for incomplete selection in the top-5% was 1.057; this resulted in higher than the median values of ORs from permuted datasets (p < 10^−4^; Fig 2). Considering the variants with ASD GWAS p < 0.1, top-5% incomplete-selection score, and ASD GWAS OR > 1, we conducted enrichment analysis for tissue-specific gene expression and for Gene Ontologies (GO) related to biological processes to gain insights regarding the molecular mechanisms involved. SNPs were assigned to the genes where they are located and/or to the nearest genes (±50KB). With a false discovery rate (FDR) < 5%, we observed significant enrichments for genes highly expressed in brain and pituitary tissues (p = 2.3*10^−5^ and p = 3*10^−5^, respectively; S3 Table). Applying a type I error rate at 5% after Bonferroni multiple testing correction, 53 GO enrichments (Table 1) were identified with the top GO result related to nervous system development (GO:0007399, p = 7.57*10^−12^). Gene sets related to the parent GO terms are reported in S4 Table. Considering the significant GO enrichments, we observed a large similarity-based network including several terms related to nervous system development (Fig 3).

**Table 1:** Gene Ontology (GO) enrichments for variants with ASD GWAS OR >1 and p < 0.1 and located in genomic regions with HB score for incomplete selection in top-5%. Parent GO are in underlined italic. Hierarchical relationships are represented by arrow symbols (→).

GO biological process	Fold Enrichment	Bonferroni P-value	P-value
*synapse organization (GO*:*0050808)*	4.09	6.76E-03	8.29E-07
→ → cellular process (GO:0009987)	1.15	1.97E-05	2.41E-09
→ single-organism cellular process (GO:0044763)	1.2	1.22E-03	1.50E-07
→ → single-organism process (GO:0044699)	1.17	9.77E-04	1.20E-07
*axon guidance (GO*:*0007411)*	3.66	1.48E-03	1.81E-07
→ axonogenesis (GO:0007409)	2.8	1.43E-02	1.75E-06
→ → axon development (GO:0061564)	2.66	2.48E-02	3.05E-06
→ → → neuron projection development (GO:0031175)	2.45	1.90E-03	2.33E-07
→ → → → cell projection organization (GO:0030030)	2.09	1.33E-03	1.63E-07
→ → → → neuron development (GO:0048666)	2.21	4.53E-03	5.55E-07
→ → → → → cell development (GO:0048468)	2.1	1.43E-07	1.76E-11
→ → → → → → cell differentiation (GO:0030154)	1.67	1.28E-07	1.57E-11
→ → → → → → → cellular developmental process (GO:0048869)	1.64	2.44E-07	2.99E-11
→ → → → → → single-organism developmental process (GO:0044767)	1.46	3.93E-06	4.81E-10
→ → → → → → → developmental process (GO:0032502)	1.44	1.46E-05	1.79E-09
→ → → → → → anatomical structure development (GO:0048856)	1.43	3.56E-05	4.37E-09
→ → → → → neuron differentiation (GO:0030182)	2.35	5.48E-06	6.73E-10
→ → → → → → generation of neurons (GO:0048699)	2.09	1.36E-06	1.66E-10
→ → → → → → → neurogenesis (GO:0022008)	2.01	5.27E-06	6.46E-10
→ → → → → → → → nervous system development (GO:0007399)	1.89	6.17E-08	7.57E-12
→ → → → → → → → → system development (GO:0048731)	1.58	3.01E-07	3.69E-11
→ → → → → → → → → → multicellular organism development (GO:0007275)	1.51	8.76E-07	1.07E-10
→ → → → → → → → → → → single-multicellular organism process (GO:0044707)	1.42	2.41E-05	2.95E-09
→ → → → → → → → → → → → multicellular organismal process (GO:0032501)	1.28	2.53E-02	3.11E-06
→ → neuron projection morphogenesis (GO:0048812)	2.67	4.66E-03	5.71E-07
→ → → cell projection morphogenesis (GO:0048858)	2.54	5.77E-05	7.08E-09
→ → → → cell morphogenesis (GO:0000902)	2.54	1.76E-07	2.16E-11
→ → → → → cellular component morphogenesis (GO:0032989)	2.42	3.84E-07	4.71E-11
→ → → → → → anatomical structure morphogenesis (GO:0009653)	1.82	3.54E-06	4.35E-10
→ → → cell part morphogenesis (GO:0032990)	2.52	5.57E-05	6.83E-09
→ → cell morphogenesis involved in neuron differentiation (GO:0048667)	2.72	7.04E-03	8.63E-07
→ → → cell morphogenesis involved in differentiation (GO:0000904)	2.92	1.81E-06	2.22E-10
→ neuron projection guidance (GO:0097485)	3.62	1.74E-03	2.13E-07
→ → movement of cell or subcellular component (GO:0006928)	1.94	2.76E-04	3.39E-08
→ → → locomotion (GO:0040011)	2.08	1.63E-04	2.00E-08
*regulation of binding (GO*:*0051098)*	3.04	7.85E-03	9.63E-07
*heart development (GO*:*0007507)*	2.71	3.03E-04	3.72E-08
→ animal organ development (GO:0048513)	1.59	2.60E-04	3.19E-08
→ cardiovascular system development (GO:0072358)	2.15	2.70E-03	3.31E-07
→ → circulatory system development (GO:0072359)	2.15	2.70E-03	3.31E-07
*pattern specification process (GO*:*0007389)*	2.61	7.79E-03	9.56E-07
*sensory organ development (GO*:*0007423)*	2.38	2.13E-02	2.61E-06
*organ morphogenesis (GO*:*0009887)*	2.09	3.74E-03	4.58E-07
*cell migration (GO*:*0016477)*	2.06	2.50E-02	3.07E-06
→ localization (GO:0051179)	1.39	1.60E-03	1.96E-07
*anatomical structure formation involved in morphogenesis (GO*:*0048646)*	2.05	1.55E-03	1.90E-07
*epithelium development (GO*:*0060429)*	1.97	2.76E-02	3.39E-06
*regulation of multicellular organismal development (GO*:*2000026)*	1.68	1.86E-02	2.29E-06
→ regulation of multicellular organismal process (GO:0051239)	1.61	4.56E-04	5.60E-08
→ regulation of developmental process (GO:0050793)	1.61	1.02E-02	1.25E-06
*regulation of localization (GO*:*0032879)*	1.54	1.88E-02	2.31E-06
*positive regulation of metabolic process (GO*:*0009893)*	1.51	5.44E-03	6.67E-07
→ positive regulation of biological process (GO:0048518)	1.36	3.58E-03	4.39E-07

To investigate our ASD results further, we considered ASD genetic correlation results available from LD Hub v1.3.1 [29] (available at http://ldsc.broadinstitute.org/). Applying a FDR at 5%, we identified eight significant correlations (S5 Table). Among these, we observed positive correlation with several advantageous traits: years of schooling (rg = 0.277, p = 2.9*10^−13^), college completion (rg = 0.339, p = 1*10^−6^), childhood intelligence (rg = 0.425, 5.74*10^−5^), openness to experience (rg = 0.421, p = 2*10^−3^).

**Fig 4:**
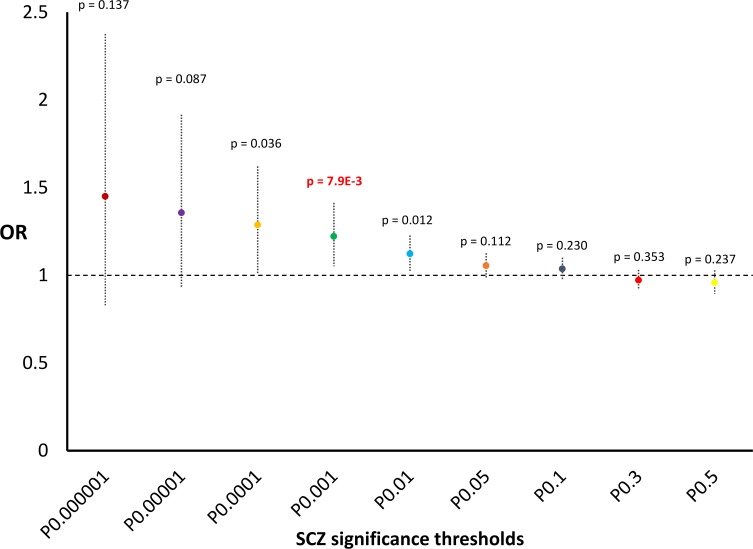
Genome-wide enrichments for scores related to incomplete selection considering different SCZ significance thresholds. Odds Ratio, 95% confidence interval, and p value are reported for each threshold investigated.

We also observed nominally significant results in relation to SCZ: a positive correlation between SCZ GWAS results and HB scores for complete selection (p = 6.37*10^−3^, S1 Table; permutation p = 3*10^−3^, S2 Fig). Although this result did not survive after multiple testing correction (see *Statistical Analysis*), we nevertheless tested enrichment for GWAS significance thresholds because this nominal result replicated previous findings obtained by independent studies with different methods [24, 25]. We observed a 22%-increased probability to be in the top-5% of scores for incomplete selection for variants with SCZ GWAS p<0.001 (OR = 1.22, 95%CI = 1.05–1.41, p = 7.9*10^−3^; Fig 4). Because the result of this subsequent analysis also did not survive Bonferroni multiple testing correction, we did not investigate the SCZ results further. No significant result was observed for the correlation analysis conducted using ADHD, BD, and MDD GWAS summary statistics (S1 Table).

## Discussion

To our knowledge, the current findings provide the first genome-wide evidence for the strong presence of natural-selection signatures in the systems genetics of ASD. Although many authors hypothesized a strong involvement of evolutionary mechanisms in ASD [4] and several studies investigated candidate genes [30]. no evidence of widespread positive selection on ASD-associated SNPs was previously reported. Using genome-wide data, we observed that common alleles associated with increased risk for ASD present a signature of positive selection in European populations. This strongly suggests that these variants have undergone positive selection during the course of human evolutionary history. Genetic correlation results support this hypothesis: ASD genetics (i.e., the set of risk variants that collectively–on a population level–influences ASD risk) strongly correlates with years of schooling, college completion, childhood intelligence, and openness to experience (S5 Table, data available at http://ldsc.broadinstitute.org/) [29]. Although these are robust genetic correlation results, further studies are needed to confirm the role of cognitive abilities in the evolutionary mechanisms involved in ASD genetics. However, multiple forms of evidence support that autism and high intelligence quotient share a diverse set of correlates, such as large brain size, fast brain growth, increased sensory and visual-spatial abilities, enhanced synaptic functions, increased attentional focus, high socioeconomic status, more deliberative decision-making, and high levels of positive assortative mating [31]. Accordingly, the genomic signatures observed in ASD risk alleles could be due to their positive associations with cognitive ability. In 2012, the Center for Disease Control and Prevention reported an ASD prevalence of 1.47% in the US population (1 in 68 children; data available at http://www.cdc.gov/ncbddd/autism/data.html). These epidemiological data can be interpreted in relation to the recent studies regarding the genetics of ASD where common genetic variation explains 49% of the ASD heritability and inherited rare and de novo mutations account for only 6% [9, 32]. Considering rare variation, the strongest contribution seems to be played by *de novo single* nucleotide variants (SNV) and copy number variants (CNV) with a reduced (but still significant) contribution from rare inherited variants [33]. In particular, rare variation associated with ASD is enrichened for disruptive truncating alleles [33–36]. Accordingly, two different evolutionary mechanisms are likely to be present in relation to ASD genetics. Rare disruptive variants predisposing to ASD are under strong purifying selection (selective removal of deleterious alleles), as already observed in certain ASD genes [37, 38]. Conversely, genetic predisposition to ASD due to common variants is highly polygenic and, taken together on a population level, these alleles present beneficial effects with respect to cognitive ability. This positive selection for ASD risk alleles increased their occurrence in human populations, and this provides a possible explanation for the disease prevalence observed by epidemiological studies. A trait related to rare alleles under strong purifying selection should present much lower prevalence. Furthermore, we observed that common ASD risk alleles with evidence of positive selection are enriched for many biological processes related to developmental mechanisms and, in particular, to mechanisms related to nervous system development. This agrees with the strong evidence indicating that the processes related to human brain development are more responsible for distinctive human traits [39]. Accordingly, ASD risk alleles could positively affect these mechanisms, causing better cognitive ability in carriers as a consequence. However, an excessive burden of these risk variants is correlated with the onset of the developmental disorders included in the autism spectrum as the evolutionary cost.

Although they did not survive multiple testing correction, the results of our SCZ analysis are consistent with two previous studies that observed enrichments for selection signatures based on inter-species comparisons (pHARs: conserved regions in non-human primates *vs*. humans; NSS: selective sweep based Neanderthals *vs*. Humans comparisons) [24, 25]. Our results suggest that natural-selection processes related to SCZ genes were also present within human evolutionary history and not only in the divergence between *Homo Sapiens* and non-human species. However, these intra-species adaptation mechanisms were particularly strong because they caused fixation of the selected allele (complete selection). We can also speculate that different evolutionary mechanisms may have acted on the genetic architecture of these two traits. Indeed, it appears that SCZ risk alleles associated with positive selection can reach fixation (complete selection) more easily than ASD risk alleles (incomplete selection).

Although SCZ GWAS has so far been more powerful than ASD (over 100 genome-wide significant SCZ loci *vs*. 1 genome-wide significant ASD locus identified; this is largely due to the sample size difference; S3 Fig), the strongest evolutionary findings were observed in ASD analysis, suggesting that natural selection acted powerfully on the systems genetics of this disorder.

As mentioned above, signatures of polygenic adaptation have been identified in the context of several phenotypic traits. However, for some of them (e.g., immune response, skin pigmentation, and anthropometric traits) single-locus signals of selection are also present. Conversely, to our knowledge no single-locus evolutionary signature has been directly related to brain functions. This can support a speculative hypothesis about the genetic architecture of brain functions. Genetic mechanisms pivotal for neural activities are strongly conserved and alleles with large effect on gene functions have an extremely high probability of being deleterious (and of consequent elimination by purifying selection). Conversely, alleles with small effects could modify the brain systems more subtly and, in some cases, provide small beneficial effects. According to our interpretation of our data, such small-effect alleles were accumulated across the genome (polygenic adaptation) to the benefit of most but to the detriment of some.

In conclusion, the present study provides evidence regarding the role of human evolution in shaping the genetic architecture of psychiatry disorders, providing a hypothesis to explain the ASD prevalence as the evolutionary cost of the polygenic adaptation of the disease risk alleles.

## Materials and methods

### Ethics statement

Publicly available GWAS summary statistics from the PGC and computational methods were used and therefore no additional ethics approval was needed. The ethics approval of PGC studies can be found in the related articles [6, 23, 40–42].

### GWAS summary statistics

We used the summary statistics of the large GWAS meta-analyses conducted on these traits by the PGC (data available at https://www.med.unc.edu/pgc/results-and-downloads) [6, 23, 40–42]. Details regarding the summary statistics used are reported in S6 Table. To exclude bias related to linkage disequilibrium (LD), we clumped the data considering the European reference panel from the 1,000 Genomes Project [43] and the following parameters: 500kb window, r^2^ < 0.25, imputation info score > 0.9, and minor allele frequency (MAF) > 0.02. For SCZ we included a single MHC single nucleotide polymorphism (SNP). To our knowledge, the PGC ASD GWAS was conducted on the largest cohort currently published and it includes samples from the Geschwind Autism Center of Excellence, the Autism Genome Project [44, 45], the Autism Genetic Resource Exchange[46, 47], the Montreal/Boston Collection [48], and the Simons Simplex Collection [49]. We were unable to identify any additional available ASD GWAS for replication.

### Hierarchical boosting algorithm

We considered natural-selection scores derived from the HB algorithm, which is a machine-learning classification framework that combines the functionality of several selection tests to uncover different genetic features expected under selective sweeps [28].

Specifically, HB is based on a machine-learning algorithm called boosting (from the mboost R package), which is a supervised algorithm that estimates linear regressions of input variables (summary statistics of selection tests) to maximize the differences between two competing scenarios (complete vs. incomplete selective sweeps). The HB method sequentially applies different boosting functions to a hierarchical classification scheme to classify optimally genomic regions into different evolutive regimes. The HB method was successfully tested with respect to simulations in a period between 10 thousand years ago (Kya) and 45 Kya, which should include both ancient and recent selective sweeps [28]. In our study, we considered two different natural-selection scenarios: complete selection (loci where a selected allele reached fixation) and incomplete selection (loci where a selected allele has not yet reached fixation). The HB method assigns a score with respect a specific genomic region. Accordingly, we assigned the HB scores to the alleles investigated in GWAS based on their locations. Since the original PGC GWAS were conducted on European populations, we considered the HB scores calculated on a European (CEU) population from the 1,000 Genomes Project.

### Statistical analysis

Statistical analysis was performed using the computing environment R (https://www.r-project.org/). For the initial correlation analysis (GWAS significance, -log_10_ p value, vs. HB score), we applied a Bonferroni correction accounting for the number of psychiatric disorders (ADHD, ASD, BP, MDD, SCZ) and the number of selection signals (incomplete-selection and complete-selection) to calculate the significance threshold adjusted for multiple testing correction (p = 5*10^−3^). For the subsequent testing of multiple GWAS significance thresholds, we applied a Bonferroni correction for the number of GWAS significance thresholds tested (ASD: 6, p = 8.3*10^−3^; SCZ: 9, p = 5.5*10^−3^). The difference in the number of GWAS significance thresholds tested is due to the difference of statistical power in the original GWAS (S3 Fig). The power analysis applied to the original ASD and SCZ GWAS was conducted using QUANTO software (http://biostats.usc.edu/Quanto.html).

To gain insights about the molecular mechanisms involved in the evolutionary pressures related to the genetics of psychiatry disorders, the results were further investigated to understand the direction of these evolutionary enrichments and to identify which tissues and biological processes are involved. SNPs were assigned to the genes where they are located and/or to the nearest genes (±50KB). DAVID v6.8 [50] (https://david.ncifcrf.gov/) was used to verify the enrichment for tissue-specific gene expression. DAVID v6.8. uses the following gene expression databases (DAVID Update Date: May 2016): Cancer Genome Anatomy Project (CGAP_EST_QUARTILE), Cancer Genome Anatomy Project Serial Analysis of Gene Expression (CGAP_SAGE_QUARTILE), U133A database of the Genomics Institute of the Novartis Research Foundation (GNF_U133A_QUARTILE), UniGene (UNIGENE_EST_QUARTILE), and Uniprot tissue (UP_TISSUE). The FDR was applied to correct the results for multiple testing [51] and q values < 0.05 were considered to be significant. Panther v11.0 [52] (http://www.pantherdb.org/) was used to conduct an overrepresentation test considering the biological processes included in the GO database for *Homo Sapiens* (released 2016-07-29). This statistical test calculates whether, considering a specific functional category, the genes included in the analysis are statistically different (overrepresented or underrepresented) from the chance expectation. Bonferroni correction for the number of tests conducted (i.e., GO for biological processes) was applied to adjust the results. To further validate our GO-enrichment results, we randomly permuted the original dataset 100 times and performed the GO-enrichment analysis on the permuted data. The number of GO enrichments observed was significantly higher than null distribution generated by the random permutations (p_permutation_ < 0.01; S4 Fig). The GO enrichment results were further investigated using REVIGO [53] (available at http://revigo.irb.hr/). Specifically, GO enrichments were used to make a graph-based visualization considering an allowed similarity of 0.7, UniProt as reference database, and Jian and Conrath method as the semantic similarity measure.

To further follow-up our findings, we used the information about genetic correlations provided by LD Hub v1.3.1 [29] (available at http://ldsc.broadinstitute.org/ldhub/). This web-tool provides information regarding genetic correlation results for 189 traits. The genetic correlations were calculated using the LD score method [54]. In our study, we applied the FDR [51] to correct the results for multiple testing and q values < 0.05 were considered to be significant.

## Supporting information

S1 Table Correlations (Spearman's rho, p value) between GWAS significance of psychiatric disorders and HB scores for incomplete and complete selection. (DOCX)

S2 Table Enrichment for incomplete-selection signals in ASD GWAS considering different thresholds. (DOCX)

S3 Table Details of the gene-expression enrichment results. (DOCX)

S4 Table Gene sets related to the parent GO terms. (DOCX)

S5 Table Genetic correlations between ASD and the phenotype traits available in the LD hub database v 1.3.1 (available at http://ldsc.broadinstitute.org/). (DOCX)

S6 Table Details of the GWAS summary statistics used in the present study. (DOCX)

S1 Fig Distribution of Spearman's rho generated from 10,000 permutations of ASD dataset. Blue lines represent the Spearman's rho observed in the real data.
(DOCX)

S2 Fig Distribution of Spearman's rho generated from 10,000 permutations of SCZ datasets. Blue lines represent the Spearman's rho observed in the real data.
(DOCX)

S3 Fig Statistical power of PGC ASD and SCZ GWAS calculated considering different thresholds, a minor allele frequency of 10%, and an effect size of OR = 1.1. The sample size for ASD and SCZ cohorts are those reported in S6 Table.
(DOCX)

S4 Fig GO-enrichment results in 100 random permutations. We report the null distribution and the frequency the GOs observed. In the ASD data, we observed enrichment for 53 GOs (p_permutation_<0.01).
(DOCX)
