# Supplementary material for: Widespread signatures of positive selection in common risk alleles associated to autism spectrum disorder
Source: PLoS Genet. 2017 Feb 10;13(2):e1006618. doi: 10.1371/journal.pgen.1006618 (PMC5328401; doi:10.1371/journal.pgen.1006618)
Supplement: S1 Table — (DOCX) [file pgen.1006618.s001.docx]

**S1 Table**: Correlations (Spearman's rho, p value) between GWAS significance of psychiatric disorders and HB scores for incomplete and complete selection.

| **Phenotypic Trait** | **Incomplete** | **Complete** |
| --- | --- | --- |
| ADHD | 0.0001, 0.7 | -0.0049, 0.039 |
| ASD | 0.0085, 3.53*10^-4^ | -0.0015, 0.526 |
| BP | 0.0041, 0.175 | 0.0011, 0.717 |
| MDD | 0.0049, 0.09 | 0.0028, 0.323 |
| SCZ | -0.0023, 0.465 | 0.0086, 6.37*10^-3^ |
