# Supplementary material for: Widespread signatures of positive selection in common risk alleles associated to autism spectrum disorder
Source: PLoS Genet. 2017 Feb 10;13(2):e1006618. doi: 10.1371/journal.pgen.1006618 (PMC5328401; doi:10.1371/journal.pgen.1006618)
Supplement: S2 Table — (DOCX) [file pgen.1006618.s002.docx]

**S2 Table**: Enrichment for incomplete-selection signals in ASD GWAS considering different thresholds.

| **Variants with incomplete-selection signature**  **(Top-5% HB score)** | | | | | | |
| --- | --- | --- | --- | --- | --- | --- |
| *GWAS thresholds* | *Observed* | *Expected* | *OR* | *95%CI* | *p* |  |
| p0.001 | 20 | 21 | 0.96 | 0.59-1.50 | 1 |  |
| p0.01 | 162 | 147 | 1.10 | 0.93-1.29 | 0.235 |  |
| p0.05 | 643 | 547 | 1.17 | 1.08-1.28 | 2.60E-04 |  |
| p0.1 | 1,107 | 927 | 1.19 | 1.11-1.28 | 9.56E-07 |  |
| p0.3 | 2,354 | 2,045 | 1.15 | 1.08-1.23 | 9.36E-06 |  |
| p0.5 | 3,204 | 2,731 | 1.17 | 1.09-1.26 | 1.74E-05 |  |
