# Supplementary material for: Widespread signatures of positive selection in common risk alleles associated to autism spectrum disorder
Source: PLoS Genet. 2017 Feb 10;13(2):e1006618. doi: 10.1371/journal.pgen.1006618 (PMC5328401; doi:10.1371/journal.pgen.1006618)
Supplement: S3 Table — (DOCX) [file pgen.1006618.s003.docx]

**S3 Table**: Details of the gene-expression enrichment results.

| **Database** | **Tissue** | **Fold**  **Enrichment** | **p** | **q** | **Genes** |
| --- | --- | --- | --- | --- | --- |
| UP_TISSUE | Brain | 1.2 | 0.000023 | 0.029 | ADCYAP1R1,AHDC1,ARID1B,AGBL4,ATP1A1,ATP11B,BET1,CXCL11,CDKN2AIPNL,CELF6,CYLD,DAOA,DDX18,DSCAM,DNAJC1,EFCAB1,EFCAB11,EFCAB12,EGFLAM,ELAVL3,EPHA4,EPHB6,ERGIC3,ETV1,EVL,FGR,GPR85,GTSE1,GLIS3,GRAMD2,GCC2,H1FOO,H2AFY,HERC4,KIAA1217,KIAA1429,KEL,MPHOSPH6,MAFB,MARVELD1,MEIS3,NGLY1,NECAB2,NDUFA4,NKX2-2,NKAIN2,OSGEPL1,ORMDL1,PKNOX2,POU2F3,PPFIBP1,PRELID2,RAB11FIP5,RAB17,RB1,RCBTB2,RYBP,RBM12,RBFOX1,RPGRIP1L,RADIL,SCOC-AS1,SESTD1,SETD5,SH3BP5L,SLITRK1,SRGAP3,SMAD7,SNRK,SUPT3H,ST3GAL3,ST8SIA3,SENP8,SMARCA2,SWI5,STARD4,STARD9,TIAM1,TBC1D32,THAP5,TPX2,TSPYL5,TRUB2,URB1,VPS39,WDR7,ANKS1A,JMJD6,ARIH1,ARMC9,ARTN,ARNTL2,ASPH,ATG3,AXIN2,ARPP19,ARPP21,CDH12,CDH2,CDH4,CDH5,CAMK2D,CAPN7,CNN1,CPA6,CTNNA2,CTNNA3,CEP250,CEP290,CRBN,CHRM5,C10orf12,C12orf29,C12orf50,C14orf180,C14orf28,C4orf22,CLDN17,CCDC172,CCDC24,CCDC80,COL5A2,COL11A1,CNTNAP2,CPNE1,CRHR2,CRAMP1,CAND1,CCNB2,CHORDC1,CDPF1,CRIM1,CYP2E1,CYP4X1,DGUOK,DGKG,DGKI,DOK5,DOK6,DRD5,DCDC2,DMRT1,DNM1,DYNC1H1,DYNC2LI1,ELMO1,ERBB4,FAM214A,FNTA,FGF12,FIBIN,FOXK1,GABRB2,GJD4,GBX2,GDNF,GRM4,GOLGA2,GDF5,GRB2,HN1L,HGSNAT,HEXA,HM13,HDAC9,IPO13,INPP4B,IFI16,IFT140,KLHL2,KIF26B,KIF2B,KIF3A,LBR,LINGO2,LRRC4C,LRRTM1,LRRTM4,LRCH3,KDM4A,MFSD11,MED23,MRAP,MAGI1,MAGI2,MFAP3L,MAP2,MRPS22,MAPK10,MAPK8IP3,MOXD1,MYO9A,MYO5A,MYLK,NELL2,NRG3,NRXN3,NCALD,NFASC,NREP,NRIP1,NAP1L5,OPALIN,OPRK1,OAF,PARVA,PMP2,PEX6,PDE1C,PLA2G4F,PLCH1,PLEKHG3,PLEKHH2,PTN,PLXND1,PODN,PARP1,GALNTL6,KCNK9,KCNH7,PDYN,PRLH,PTGS1,PRKAB1,PRKDC,PPP1R17,PPP2R5E,PPP2R2B,PTPRF,PTPRM,POMK,PCDH17,PCDH8,PKM,RHOJ,ROMO1,ROR1,RGS9,RHBDD1,RNF111,RYR1,RYR3,SCART1,SEZ6L,SZT2,STK32B,SPRN,SCOC,SDK1,SPCS3,SLC12A2,SLC20A2,SLC27A4,SLC39A10,SLC6A9,SLC8A2,SORL1,SYNE2,SPECC1L,SCP2,SYCE1,SNTG1,SNCA,TCP11,TACR3,TENM2,TNS1,TSPAN16,TTC26,TTC38,TMX3,TBXAS1,TLL2,TRAPPC9,TMCO5A,TMEM192,TMEM200A,TMEM242,TMEM87A,TDO2,TPRG1,TWIST1,UQCC1,UBE2B,UBE2V2,UNC80,LOC151121,VRK3,VCAN,VWA3A,ZFYVE27,ZNF300,ZNF436,ZNF462,ZNF473,ZNF507,ZNF510,ZNF608,ZNF653 |
| GNF_U133A_QUARTILE | Pituitary_3rd | 1.5 | 0.00003 | 0.032 | AKTIP,AHDC1,BCL2L1,BEND5,CYLD,DSCAM,ELAVL3,EPHA4,EPHB6,ETV5,FYB,KIAA1217,MPL,NECAB2,NDUFA4,NKX2-2,PKNOX2,RBFOX1,ARHGAP26,SKP1,SRGAP3,ST8SIA3,SMARCA2,TIAM1,WDR7,XRCC4,ALCAM,ACOT7,ADGRL3,ANK2,ARMC9,ARTN,ARPP19,ARPP21,CDH10,CDH12,CDH2,CDH4,CTNNA2,CDC14B,CEP290,CHRM5,CNTNAP2,CDKL3,CYP2E1,CYP3A43,DGKI,DNM1,DYNC1H1,ELMO1,ERBB4,ESR2,FGF1,FGF12,GALC,GRM4,GNMT,HOXC13,HIPK2,IPO13,IFT122,ICA1,KALRN,KIF3A,MFSD11,MAGI1,MAGI2,MFAP3L,MAP2,MAPK10,MAPK8IP3,MYO9A,MYLK,NELL2,NRXN3,NCALD,NFASC,NREP,NFIB,PMP2,PDE1C,PLCH1,PSAT1,PLEKHG3,PTN,PPP2R2B,PCDH17,PCDH8,PDP1,RHO,RYR3,SCARB2,SFRP5,SEZ6L,SLC12A2,SLC16A7,SLC8A2,SORL1,SRD5A3,SNCA,TTC38,TCF7,TRPV5,TDO2,UBE2B,UTRN,ZBTB20 |
