# Supplementary material for: Widespread signatures of positive selection in common risk alleles associated to autism spectrum disorder
Source: PLoS Genet. 2017 Feb 10;13(2):e1006618. doi: 10.1371/journal.pgen.1006618 (PMC5328401; doi:10.1371/journal.pgen.1006618)
Supplement: S4 Table — (DOCX) [file pgen.1006618.s004.docx]

**S4 Table**: Gene sets related to the parent GO terms.

| **GO term** | **Genes** |
| --- | --- |
| synapse organization | SDK1, GDNF, FNTA, CDH2, ETV5, PCDH17, DSCAM, PLXND1, SNCA, LPHN3, UTRN, MYO5A, MAGI2, NRXN3, LRRTM4, NFASC, LRRTM1, SEZ6L |
| axon guidance | GRB2, EPYC, ETV1, ALCAM, PLXND1, EVL, ENAH, FYN, DCC, ARTN, MAPK8IP3, PTPRM, NFIB, SPTB, KIF26B, ROBO1, GBX2, CDH4, TENM2, EPHA4, NRXN3, NELL2, NFASC |
| regulation of binding | CPNE1, GOLGA2, GTSE1, TIAM1, BMP4, EPHB6, AKTIP, PITX2, PLXND1, IFI16, PTPRF, SORL1, NFIB, RB1, ICA1, PARP1, ZNF462, ID1, CRBN, PINX1, HIPK2, PPARA, EGF, EPHA4, AURKA, TWIST1 |
| heart development | AXIN2, C3orf58, COL11A1, SNAI2, FGF12, OSR1, XIRP2, BMP4, IFT140, PARVA, PITX2, SALL1, PLXND1, TBC1D32, PRKDC, JMJD6, GRHL2, RPGRIP1L, PPARD, IFT122, SOX17, HDAC9, ERBB4, ANK2, RYR1, ID1, PTN, SMAD7, ROBO1, PPARA, HIF1A, BVES, CAMK2D, PROX1, MYLK2, NELL2, TRPS1, TWIST1, KIF3A |
| pattern specification process | AXIN2, NRG3, OSR1, PAX1, NKX2-2, RFX3, BMP4, PCDH8, HOXC13, IFT140, ASPH, CELSR1, PITX2, PLXND1, TBC1D32, PRKDC, RPGRIP1L, IFT122, RNF111, SOX17, MAFB, ERBB4, HIPK2, GBX2, HIF1A, CYP26B1, EGF, DMRT3, AURKA, NELL2, DYNC2LI1, MEOX2, KIF3A |
| sensory organ development | IL4, SDK1, COL11A1, OSR1, RHOJ, BMP4, COL5A2, HOXC13, IFT140, CELSR1, DSCAM, PITX2, SALL1, TBC1D32, MAF, BNC2, JMJD6, GRHL2, RPGRIP1L, GABRB2, KERA, IFT122, PTPRM, MAFB, CEP290, USH2A, PTN, HIPK2, GBX2, RHO, HIF1A, CYP26B1, PROX1, FOXL2, TWIST1, KIF3A |
| organ morphogenesis | AXIN2, SDK1, NRG3, COL11A1, SNAI2, OSR1, PAX1, GDNF, XIRP2, FGF1, BMP4, COL5A2, MYLK, HOXC13, CDH2, IFT140, PARVA, ARG1, CELSR1, DSCAM, PITX2, SALL1, PLXND1, EVL, GRHL2, IFT122, SOX17, MAPK8IP3, PTPRM, MAFB, NFIB, CEP290, CTNNA2, LAMB1, KIF26B, RYR1, ID1, PTN, HIPK2, SMAD7, PPARA, GBX2, HIF1A, CYP26B1, MYO5A, TCF7, ACTG2, PROX1, MYLK2, NELL2, FOXL2, TRPS1, TWIST1, KIF3A |
| cell migration | ADAMTS12, ANKS1A, CXCL1, GRB2, NRG3, SNAI2, GDNF, TIAM1, CXCL11, CDH2, CD84, PARVA, CELSR1, PITX2, PLXND1, ELMO1, FYN, CXCL5, PODXL, PTPRF, PPARD, SOX17, DCC, FGR, ARTN, ERBB4, CTNNA2, VCAN, ANGPT2, LAMB1, PPBP, LPHN3, TNS1, ID1, DCDC2, ROBO1, SYNE2, GBX2, HIF1A, DMRT1, BVES, CCDC141, EPHA4, POMK, PF4, PROX1, NELL2, TWIST1 |
| anatomical structure formation involved in morphogenesis | AXIN2, SDK1, UBE2B, GRB2, SOX7, NRG3, COL11A1, SNAI2, OSR1, PAX1, GDNF, FGF1, RFX3, BMP4, PCDH8, IFT140, PARVA, CELSR1, DSCAM, PITX2, SALL1, PLXND1, ONECUT1, PRKDC, JMJD6, GRHL2, TTC26, RPGRIP1L, BBS10, PODXL, ACKR3, IFT122, CEP250, SOX17, PTPRM, NFIB, CEP290, ANGPT2, LAMB1, ANK2, KIF26B, ID1, HIPK2, ROBO1, PPARA, GBX2, HIF1A, FZD8, EGF, MYPN, NRXN3, PROX1, NELL2, NFASC, TRPS1, DYNC2LI1, RAB17, TWIST1, MEOX2, KIF3A |
| epithelium development | AXIN2, GRB2, SNAI2, OSR1, PAX1, GDNF, NKX2-2, FGF1, RFX3, BMP4, PCDH8, HOXC13, ESR2, CDH2, IFT140, CELSR1, PITX2, SALL1, PLXND1, TBC1D32, ONECUT1, MAF, PRKDC, GRHL2, CRHR2, RPGRIP1L, PODXL, IFT122, SOX17, ARHGAP12, NFIB, CEP290, USH2A, ERBB4, KIF26B, LCE1B, ID1, PTN, SMAD7, GBX2, HIF1A, DMRT1, MAGI2, CYP26B1, EGF, FZD7, EPHA4, TCF7, PROX1, FOXL2, TWIST1, MEOX2, KIF3A |
| regulation of multicellular organismal development | IL4, AXIN2, ADAMTS12, SDK1, CPNE1, IL15, CYSLTR2, NRG3, H2AFY2, SNAI2, PRKCH, OSR1, NREP, GDNF, NKX2-2, TIAM1, FGF1, EPYC, EEF2K, RFX3, BMP4, COL5A2, CDH2, ROR1, ETV5, CELSR1, DSCAM, PLXND1, MAF, LINGO2, UBE2V2, GDF5, GRHL2, FYN, H2AFY, CDH5, GJD4, CDC20, SLITRK1, PTPRF, SFRP5, PPARD, IFT122, KEL, SOX17, SORL1, C6, DCC, PTPRM, MAFB, LRRC4C, NFIB, CYLD, HDAC9, ERBB4, RB1, ANGPT2, RBFOX1, LPHN3, TLL2, PPP2CA, ID1, PTN, HIPK2, SMAD7, ROBO1, CDH4, HIF1A, DMRT1, MAGI2, CYP26B1, EGF, DGUOK, DMRT3, FZD7, EPHA4, PF4, NRXN3, PROX1, LRRTM1, TRPS1, RAB17, TWIST1, MEOX2, HOOK3 |
| regulation of localization | IL4, AXIN2, CAPN7, TRPV6, TLR10, CXCL1, FFAR4, PODN, PTGS1, NRG3, ATP1A1, NKD2, SNAI2, NKAIN2, FGF12, GTSE1, GDNF, SCP2, SARAF, TIAM1, FGF1, KCNU1, EEF2K, RFX3, BMP4, MYLK, VDAC3, CXCL11, CDH2, CD84, ARG1, PCDH17, ASPH, DSCAM, PITX2, PLXND1, EVL, CYP4A11, ONECUT1, WDR75, SNCA, GCC2, RYR3, FYN, H2AFY, CXCL5, CRHR2, PODXL, IRS1, RHBDD1, SFRP5, PPARD, NUP54, SPIDR, KEL, SORL1, FGR, SRGAP3, PTPRM, CEP290, ABHD5, RAB11FIP5, CYLD, HDAC9, MSR1, ERBB4, OLFM4, RB1, KIAA0226, ANGPT2, LAMB1, ICA1, ADCYAP1R1, ANK2, PPBP, PARP1, RYR1, CRBN, ABCG5, PINX1, PTN, CTNNA3, GHRHR, SMAD7, ROBO1, SYNE2, UTRN, PPARA, OPRK1, DYRK2, HIF1A, DNM1, MAGI2, BVES, EGF, MYO5A, FZD7, CAMK2D, BCL2L1, KCNH7, PF4, DNAJC1, PROX1, MYLK2, LRRTM1, SLAMF1, AIM2, FOXL2, RAB17, TWIST1, MEOX2, KIF3A, ARPP19 |
| positive regulation of metabolic process | MEIS3, PRKAB1, IL4, AXIN2, UBE2B, SKP1, IL15, POU2F3, HNRNPR, CYSLTR2, GRB2, FFAR4, SOX7, NRG3, NKD2, SNAI2, GOLGA2, GLIS1, OSR1, PAX1, GDNF, FNTA, NKX2-2, SCP2, CEBPD, PYURF, STARD4, TIAM1, FGF1, DRD5, RFX3, BMP4, HOXC13, ESR2, CXCL11, CDH2, ETV1, ETV5, ASPH, DSCAM, VRK3, AKTIP, PITX2, SALL1, UQCC1, ONECUT1, MAF, UBE2V2, PRKDC, SNCA, ARID1B, GDF5, GRHL2, FYN, H2AFY, CRHR2, CDC20, MAPK10, IFI16, NFE2L3, IRS1, RHBDD1, ACKR3, PPARD, FOXK1, SPIDR, RNF111, SOX17, SORL1, C6, TAF4, ARIH1, FGR, MAPK8IP3, MAFB, NFIB, CAND1, CEP290, TSPYL5, ABHD5, NOBOX, BCLAF1, ERBB4, RB1, ADCYAP1R1, ANK2, SUPT3H, PARP1, APOBEC1, CDC14B, PPP2CA, ZNF462, ID1, SLC39A10, HIPK2, GHRHR, MAP3K7, TPX2, SMAD7, SMARCA2, ROBO1, PPARA, GBX2, OPRK1, DYRK2, GRM4, HIF1A, DMRT1, EPHX2, FZD8, MAGI2, BVES, CYP26B1, EGF, GLIS3, NRIP1, FZD7, CAMK2D, EPHA4, AURKA, PF4, ACTG2, PROX1, ARNTL2, MYLK2, SLAMF1, MRAP, AIM2, FOXL2, TRPS1, TWIST1, ROMO1, MEOX2, ARPP19 |
