# Supplementary material for: Widespread signatures of positive selection in common risk alleles associated to autism spectrum disorder
Source: PLoS Genet. 2017 Feb 10;13(2):e1006618. doi: 10.1371/journal.pgen.1006618 (PMC5328401; doi:10.1371/journal.pgen.1006618)
Supplement: S5 Table — (DOCX) [file pgen.1006618.s005.docx]

**S5 Table**: Genetic correlations between ASD and 189 phenotype traits available in the LD hub database v1.3.1 (available at <http://ldsc.broadinstitute.org/>).

| **Trait** | **rg** | **se** | **z** | **p** | **q** |
| --- | --- | --- | --- | --- | --- |
| Years of schooling | 0.277 | 0.038 | 7.299 | 2.90E-13 | 5.48E-11 |
| PGC cross-disorder analysis | 0.363 | 0.06 | 6.03 | 1.64E-09 | 1.55E-07 |
| College completion | 0.339 | 0.069 | 4.892 | 1.00E-06 | 6.30E-05 |
| Childhood_IQ | 0.425 | 0.106 | 4.023 | 5.74E-05 | 0.002712 |
| Subjective well being | -0.255 | 0.067 | -3.804 | 1.42E-04 | 0.005368 |
| Schizophrenia | 0.181 | 0.051 | 3.514 | 4.41E-04 | 0.013892 |
| Phospholipids in large HDL | -0.324 | 0.098 | -3.291 | 0.001 | 0.027 |
| Neo-openness to experience | 0.421 | 0.136 | 3.085 | 0.002 | 0.04725 |
| Concentration of large HDL particles | -0.284 | 0.097 | -2.931 | 0.003 | 0.0567 |
| Cholesterol esters in medium VLDL | 0.268 | 0.089 | 3.012 | 0.003 | 0.0567 |
| Free cholesterol in medium HDL | -0.405 | 0.141 | -2.879 | 0.004 | 0.068727 |
| Phospholipids in medium HDL | -0.392 | 0.144 | -2.729 | 0.006 | 0.0945 |
| Total lipids in large HDL | -0.261 | 0.097 | -2.675 | 0.007 | 0.0945 |
| Cholesterol esters in large VLDL | 0.228 | 0.085 | 2.68 | 0.007 | 0.0945 |
| Total cholesterol in medium VLDL | 0.238 | 0.092 | 2.591 | 0.01 | 0.1155 |
| Total lipids in small VLDL | 0.244 | 0.094 | 2.587 | 0.01 | 0.1155 |
| Total cholesterol in HDL | -0.287 | 0.112 | -2.558 | 0.011 | 0.1155 |
| Concentration of medium HDL particles | -0.398 | 0.156 | -2.549 | 0.011 | 0.1155 |
| Total lipids in medium HDL | -0.405 | 0.162 | -2.494 | 0.013 | 0.12285 |
| Concentration of small VLDL particles | 0.229 | 0.092 | 2.479 | 0.013 | 0.12285 |
| Concentration of very large VLDL particles | 0.296 | 0.12 | 2.47 | 0.014 | 0.126 |
| Free cholesterol in small VLDL | 0.25 | 0.105 | 2.382 | 0.017 | 0.146046 |
| Concentration of large VLDL particles | 0.223 | 0.095 | 2.355 | 0.019 | 0.15613 |
| Total lipids in very large VLDL | 0.24 | 0.103 | 2.326 | 0.02 | 0.1575 |
| Total cholesterol in small VLDL | 0.26 | 0.112 | 2.317 | 0.021 | 0.15876 |
| Total lipids in medium VLDL | 0.214 | 0.094 | 2.28 | 0.023 | 0.167192 |
| Free cholesterol in large HDL | -0.246 | 0.11 | -2.244 | 0.025 | 0.175 |
| fasting glucose main effect | -0.157 | 0.07 | -2.23 | 0.026 | 0.1755 |
| Triglycerides in small VLDL | 0.22 | 0.101 | 2.18 | 0.029 | 0.189 |
| Isoleucine | 0.242 | 0.113 | 2.142 | 0.032 | 0.195097 |
| Total cholesterol in medium HDL | -0.344 | 0.161 | -2.141 | 0.032 | 0.195097 |
| extreme height | -0.13 | 0.063 | -2.067 | 0.039 | 0.230344 |
| Total lipids in small HDL | -0.341 | 0.168 | -2.033 | 0.042 | 0.240546 |
| Cholesterol esters in medium HDL | -0.339 | 0.169 | -2.008 | 0.045 | 0.250147 |
| Total cholesterol in large VLDL | 0.194 | 0.099 | 1.952 | 0.051 | 0.252 |
| Phospholipids in small VLDL | 0.193 | 0.099 | 1.954 | 0.051 | 0.252 |
| Glucose | -0.207 | 0.107 | -1.933 | 0.053 | 0.252 |
| Mean diameter for VLDL particles | 0.192 | 0.1 | 1.925 | 0.054 | 0.252 |
| height_2010 | -0.082 | 0.043 | -1.916 | 0.055 | 0.252 |
| Triglycerides in very large VLDL | 0.193 | 0.101 | 1.918 | 0.055 | 0.252 |
| serum cystatin c | 0.141 | 0.074 | 1.911 | 0.056 | 0.252 |
| Concentration of medium VLDL particles | 0.177 | 0.093 | 1.909 | 0.056 | 0.252 |
| Mean diameter for HDL particles | -0.207 | 0.109 | -1.897 | 0.058 | 0.252 |
| child birth length | 0.195 | 0.103 | 1.888 | 0.059 | 0.252 |
| Total cholesterol in large HDL | -0.21 | 0.112 | -1.883 | 0.06 | 0.252 |
| Total lipids in large VLDL | 0.184 | 0.101 | 1.822 | 0.068 | 0.277941 |
| Age at Menopause | 0.108 | 0.06 | 1.807 | 0.071 | 0.277941 |
| Age at Menarche | -0.079 | 0.044 | -1.792 | 0.073 | 0.277941 |
| Free cholesterol in large VLDL | 0.164 | 0.091 | 1.795 | 0.073 | 0.277941 |
| Triglycerides in chylomicrons and largest VLDL particles | 0.192 | 0.108 | 1.788 | 0.074 | 0.277941 |
| Free cholesterol in medium VLDL | 0.174 | 0.098 | 1.778 | 0.075 | 0.277941 |
| Neuroticism | 0.149 | 0.085 | 1.749 | 0.08 | 0.287 |
| Leucine | 0.279 | 0.16 | 1.745 | 0.081 | 0.287 |
| Cholesterol esters in large HDL | -0.178 | 0.102 | -1.738 | 0.082 | 0.287 |
| Serum total triglycerides | 0.15 | 0.09 | 1.669 | 0.095 | 0.326455 |
| Apolipoprotein B | 0.206 | 0.126 | 1.63 | 0.103 | 0.347625 |
| waist-to-hip ratio | 0.075 | 0.047 | 1.587 | 0.113 | 0.374684 |
| Phospholipids in very large HDL | -0.186 | 0.118 | -1.576 | 0.115 | 0.374741 |
| Concentration of very large HDL particles | -0.216 | 0.14 | -1.538 | 0.124 | 0.39722 |
| urinary albumin-to-creatinine ratio (non-diabetes) | -0.168 | 0.113 | -1.492 | 0.136 | 0.4284 |
| Triglycerides in medium VLDL | 0.155 | 0.106 | 1.473 | 0.141 | 0.435 |
| Neo-conscientiousness | -0.255 | 0.175 | -1.46 | 0.144 | 0.435 |
| Depressive symptoms | 0.111 | 0.076 | 1.458 | 0.145 | 0.435 |
| Phospholipids in very large VLDL | 0.148 | 0.104 | 1.421 | 0.155 | 0.457734 |
| Triglycerides in large VLDL | 0.137 | 0.098 | 1.395 | 0.163 | 0.469875 |
| Phospholipids in medium VLDL | 0.136 | 0.098 | 1.39 | 0.165 | 0.469875 |
| Citrate | -0.18 | 0.13 | -1.382 | 0.167 | 0.469875 |
| Glycoprotein acetyls, mainly a1-acid glycoprotein | 0.152 | 0.112 | 1.36 | 0.174 | 0.469875 |
| Mean Accumbens | -0.232 | 0.171 | -1.355 | 0.175 | 0.469875 |
| Major depressive disorder | 0.135 | 0.1 | 1.354 | 0.176 | 0.469875 |
| Rheumatoid Arthritis | -0.077 | 0.057 | -1.348 | 0.178 | 0.469875 |
| Phospholipids in large VLDL | 0.132 | 0.099 | 1.343 | 0.179 | 0.469875 |
| Triglycerides in very small VLDL | 0.126 | 0.096 | 1.313 | 0.189 | 0.479961 |
| Coronary artery disease | -0.063 | 0.049 | -1.306 | 0.191 | 0.479961 |
| Free cholesterol in very large HDL | -0.203 | 0.155 | -1.305 | 0.192 | 0.479961 |
| Albumin | -0.202 | 0.155 | -1.302 | 0.193 | 0.479961 |
| adiponectin | -0.135 | 0.105 | -1.28 | 0.201 | 0.493364 |
| Total lipids in chylomicrons and largest VLDL particles | 0.139 | 0.111 | 1.25 | 0.211 | 0.511269 |
| Apolipoprotein A-I | -0.159 | 0.129 | -1.228 | 0.22 | 0.526329 |
| Ever vs never smoked | 0.087 | 0.072 | 1.21 | 0.226 | 0.533925 |
| 22:6, docosahexaenoic acid | 0.114 | 0.105 | 1.079 | 0.281 | 0.649976 |
| Lung cancer (all) | -0.109 | 0.101 | -1.077 | 0.282 | 0.649976 |
| Asthma | -0.114 | 0.108 | -1.053 | 0.292 | 0.664916 |
| HbA1C | 0.102 | 0.1 | 1.021 | 0.307 | 0.69075 |
| Total lipids in very large HDL | -0.146 | 0.145 | -1.009 | 0.313 | 0.695965 |
| Attention deficit hyperactivity disorder | -0.13 | 0.13 | -0.999 | 0.318 | 0.698861 |
| Phospholipids in medium LDL | 0.116 | 0.125 | 0.927 | 0.354 | 0.769035 |
| serumurate_overweight_all | -0.065 | 0.072 | -0.909 | 0.363 | 0.777236 |
| Mean Pallidum | -0.105 | 0.116 | -0.904 | 0.366 | 0.777236 |
| Mean platelet volume | -0.069 | 0.077 | -0.887 | 0.375 | 0.784452 |
| waist circumference | 0.039 | 0.044 | 0.876 | 0.381 | 0.784452 |
| Chronic Kidney Disease | -0.123 | 0.142 | -0.867 | 0.386 | 0.784452 |
| serum creatinine (non-diabetes) | -0.055 | 0.064 | -0.867 | 0.386 | 0.784452 |
| lumbar spine bone mineral density | 0.055 | 0.065 | 0.846 | 0.397 | 0.789124 |
| Childhood obesity | -0.058 | 0.069 | -0.84 | 0.401 | 0.789124 |
| Total lipids in very small VLDL | 0.092 | 0.11 | 0.835 | 0.404 | 0.789124 |
| Triglycerides | 0.053 | 0.063 | 0.833 | 0.405 | 0.789124 |
| Bipolar disorder | 0.063 | 0.077 | 0.82 | 0.412 | 0.792273 |
| Former vs Current smoker | 0.088 | 0.108 | 0.814 | 0.415 | 0.792273 |
| Glutamine | -0.107 | 0.134 | -0.8 | 0.424 | 0.79904 |
| ICV | -0.076 | 0.095 | -0.794 | 0.427 | 0.79904 |
| platelet count | 0.048 | 0.062 | 0.782 | 0.434 | 0.801433 |
| femoral neck bone mineral density | 0.041 | 0.054 | 0.772 | 0.44 | 0.801433 |
| Phospholipids in chylomicrons and largest VLDL particles | 0.088 | 0.114 | 0.77 | 0.441 | 0.801433 |
| Mean Putamen | -0.051 | 0.069 | -0.736 | 0.462 | 0.8316 |
| 18:2, linoleic acid (LA) | 0.086 | 0.119 | 0.722 | 0.47 | 0.8316 |
| Crohn's disease | -0.034 | 0.047 | -0.72 | 0.471 | 0.8316 |
| HOMA-IR | -0.087 | 0.123 | -0.711 | 0.477 | 0.8316 |
| urinary albumin-to-creatinine ratio | -0.082 | 0.117 | -0.703 | 0.482 | 0.8316 |
| Phospholipids in IDL | -0.105 | 0.15 | -0.7 | 0.484 | 0.8316 |
| Cigarettes smoked per day | -0.082 | 0.119 | -0.693 | 0.489 | 0.832622 |
| Concentration of very small VLDL particles | 0.073 | 0.106 | 0.682 | 0.495 | 0.835313 |
| Free cholesterol in IDL | -0.091 | 0.136 | -0.67 | 0.503 | 0.840553 |
| Ratio of bisallylic groups to double bonds | -0.051 | 0.077 | -0.663 | 0.507 | 0.840553 |
| Type 2 Diabetes | -0.048 | 0.078 | -0.618 | 0.537 | 0.874525 |
| Total cholesterol in small LDL | 0.086 | 0.139 | 0.618 | 0.537 | 0.874525 |
| obesity class 3 | 0.057 | 0.095 | 0.604 | 0.546 | 0.874525 |
| Infant head circumference | 0.063 | 0.104 | 0.604 | 0.546 | 0.874525 |
| Parkinson's disease | 0.052 | 0.087 | 0.591 | 0.555 | 0.881471 |
| Total cholesterol in medium LDL | 0.076 | 0.133 | 0.574 | 0.566 | 0.885 |
| Free cholesterol in large LDL | -0.079 | 0.138 | -0.571 | 0.568 | 0.885 |
| Inflammatory Bowel Disease (Euro) | -0.026 | 0.047 | -0.557 | 0.578 | 0.885 |
| obesity class 1 | 0.03 | 0.054 | 0.548 | 0.583 | 0.885 |
| acetate | 0.081 | 0.149 | 0.545 | 0.586 | 0.885 |
| HOMA-B | 0.06 | 0.11 | 0.544 | 0.587 | 0.885 |
| Leptin_adjBMI | 0.056 | 0.105 | 0.538 | 0.59 | 0.885 |
| Total cholesterol in very large HDL | -0.095 | 0.181 | -0.524 | 0.6 | 0.892913 |
| fasting proinsulin | 0.057 | 0.113 | 0.503 | 0.615 | 0.908086 |
| child birth weight | 0.044 | 0.089 | 0.494 | 0.621 | 0.909837 |
| age of smoking initiation | -0.07 | 0.148 | -0.474 | 0.635 | 0.912068 |
| Hip circumference | 0.022 | 0.046 | 0.474 | 0.635 | 0.912068 |
| serum creatinine | -0.03 | 0.064 | -0.472 | 0.637 | 0.912068 |
| Valine | 0.058 | 0.129 | 0.449 | 0.654 | 0.929368 |
| Total cholesterol in LDL | 0.058 | 0.135 | 0.434 | 0.664 | 0.936537 |
| obesity class 2 | 0.027 | 0.064 | 0.423 | 0.672 | 0.9408 |
| LDL cholesterol | -0.024 | 0.059 | -0.403 | 0.687 | 0.949079 |
| Description of average fatty acid chain length, not actual carbon number | 0.049 | 0.124 | 0.395 | 0.693 | 0.949079 |
| Body fat | 0.026 | 0.067 | 0.39 | 0.696 | 0.949079 |
| Alzheimer's disease | 0.042 | 0.109 | 0.388 | 0.698 | 0.949079 |
| Concentration of small LDL particles | 0.046 | 0.123 | 0.371 | 0.71 | 0.950287 |
| Leptin_not_adjBMI | -0.033 | 0.091 | -0.364 | 0.716 | 0.950287 |
| extreme waist-to-hip ratio | 0.04 | 0.11 | 0.361 | 0.718 | 0.950287 |
| Lung cancer (squamous cell) | -0.052 | 0.144 | -0.36 | 0.719 | 0.950287 |
| Omega-3 fatty acids | 0.035 | 0.103 | 0.344 | 0.731 | 0.954124 |
| Acetoacetate | 0.044 | 0.128 | 0.342 | 0.732 | 0.954124 |
| Total Cholesterol | 0.016 | 0.049 | 0.331 | 0.741 | 0.959047 |
| Serum total cholesterol | -0.044 | 0.137 | -0.318 | 0.75 | 0.959047 |
| Phospholipids in large LDL | -0.043 | 0.135 | -0.317 | 0.751 | 0.959047 |
| Total lipids in small LDL | 0.04 | 0.13 | 0.308 | 0.758 | 0.96149 |
| fasting insulin main effect | 0.025 | 0.088 | 0.289 | 0.773 | 0.970033 |
| Heart rate | 0.018 | 0.062 | 0.286 | 0.775 | 0.970033 |
| Mean Hippocampus | -0.034 | 0.133 | -0.257 | 0.797 | 0.981305 |
| Mean Caudate | 0.021 | 0.085 | 0.246 | 0.805 | 0.981305 |
| Concentration of large LDL particles | -0.034 | 0.14 | -0.245 | 0.806 | 0.981305 |
| Ratio of bisallylic groups to total fatty acids | 0.019 | 0.082 | 0.235 | 0.815 | 0.981305 |
| Triglycerides in very large HDL | 0.024 | 0.11 | 0.22 | 0.826 | 0.981305 |
| Free cholesterol | -0.037 | 0.173 | -0.212 | 0.832 | 0.981305 |
| extreme bmi | 0.016 | 0.075 | 0.21 | 0.834 | 0.981305 |
| Total lipids in large LDL | -0.028 | 0.14 | -0.203 | 0.839 | 0.981305 |
| Alanine | -0.022 | 0.115 | -0.194 | 0.846 | 0.981305 |
| Mean diameter for LDL particles | -0.028 | 0.152 | -0.187 | 0.852 | 0.981305 |
| Mean Thalamus | -0.025 | 0.142 | -0.177 | 0.859 | 0.981305 |
| Concentration of IDL particles | -0.025 | 0.137 | -0.178 | 0.859 | 0.981305 |
| Triglycerides in small HDL | 0.021 | 0.127 | 0.166 | 0.868 | 0.981305 |
| Concentration of chylomicrons and largest VLDL particles | 0.019 | 0.115 | 0.162 | 0.872 | 0.981305 |
| Anorexia Nervosa | 0.009 | 0.06 | 0.145 | 0.884 | 0.981305 |
| 2hr glucose adjusted for BMI | 0.018 | 0.126 | 0.146 | 0.884 | 0.981305 |
| HDL cholesterol | 0.008 | 0.055 | 0.142 | 0.887 | 0.981305 |
| Forearm Bone mineral density | -0.021 | 0.153 | -0.14 | 0.889 | 0.981305 |
| Average number of methylene groups per a double bond | -0.013 | 0.092 | -0.138 | 0.891 | 0.981305 |
| Triglycerides in IDL | -0.015 | 0.113 | -0.131 | 0.896 | 0.981305 |
| Eczema | -0.014 | 0.108 | -0.128 | 0.898 | 0.981305 |
| Phospholipids in very small VLDL | -0.015 | 0.128 | -0.12 | 0.904 | 0.981305 |
| Mono-unsaturated fatty acids | 0.014 | 0.123 | 0.117 | 0.907 | 0.981305 |
| Total lipids in IDL | -0.015 | 0.138 | -0.106 | 0.915 | 0.981305 |
| Total cholesterol in large LDL | -0.014 | 0.131 | -0.103 | 0.918 | 0.981305 |
| Average number of double bonds in a fatty acid chain | 0.01 | 0.096 | 0.102 | 0.919 | 0.981305 |
| body mass index | 0.004 | 0.05 | 0.088 | 0.93 | 0.987472 |
| Omega-9 and saturated fatty acids | 0.01 | 0.132 | 0.076 | 0.94 | 0.988141 |
| Concentration of medium LDL particles | 0.01 | 0.135 | 0.071 | 0.944 | 0.988141 |
| Cholesterol esters in medium LDL | 0.009 | 0.135 | 0.067 | 0.947 | 0.988141 |
| Total lipids in medium LDL | 0.008 | 0.134 | 0.059 | 0.953 | 0.988141 |
| Creatinine | 0.005 | 0.098 | 0.051 | 0.959 | 0.988141 |
| Tyrosine | 0.006 | 0.119 | 0.047 | 0.962 | 0.988141 |
| Ulcerative colitis | 0.003 | 0.066 | 0.039 | 0.969 | 0.989951 |
| overweight | 0.001 | 0.056 | 0.021 | 0.983 | 0.991245 |
| Free cholesterol to esterified cholesterol ratio | -0.004 | 0.184 | -0.022 | 0.983 | 0.991245 |
| Total cholesterol in IDL | -0.002 | 0.139 | -0.018 | 0.986 | 0.991245 |
| Phenylalanine | 0.001 | 0.126 | 0.01 | 0.992 | 0.992 |
