# Supplementary material for: Widespread signatures of positive selection in common risk alleles associated to autism spectrum disorder
Source: PLoS Genet. 2017 Feb 10;13(2):e1006618. doi: 10.1371/journal.pgen.1006618 (PMC5328401; doi:10.1371/journal.pgen.1006618)
Supplement: S6 Table — (DOCX) [file pgen.1006618.s006.docx]

**S6 Table**: Details of the GWAS summary statistics used in the present study.

| **Phenotypic Trait** | **Sample size** | **Reference** |
| --- | --- | --- |
| Attention Deficit Hyperactivity Disorder  (ADHD) | 2,064 trios, 896 cases, and 2,455 controls | 27 |
| Autism Spectrum Disorder  (ASD) | 5,305 ASD cases and 5,305 pseudocontrols | 7 |
| Bipolar Disorder  (BP) | 11,974 cases and 51,792 controls | 26 |
| Major Depressive Disorder  (MDD) | 9,240 cases and 9,519 controls | 28 |
| Schizophrenia  (SCZ) | 36,989 cases and 113,075 controls | 23 |
