# Supplementary material for: Widespread signatures of positive selection in common risk alleles associated to autism spectrum disorder
Source: PLoS Genet. 2017 Feb 10;13(2):e1006618. doi: 10.1371/journal.pgen.1006618 (PMC5328401; doi:10.1371/journal.pgen.1006618)
Supplement: S1 Fig — Blue lines represent the Spearman's rho observed in the real data. (DOCX) [file pgen.1006618.s007.docx]

**S1 Fig.**: Distribution of Spearman's rho generated from 10,000 permutations of ASD dataset. Blue lines represent the Spearman's rho observed in the real data.

**
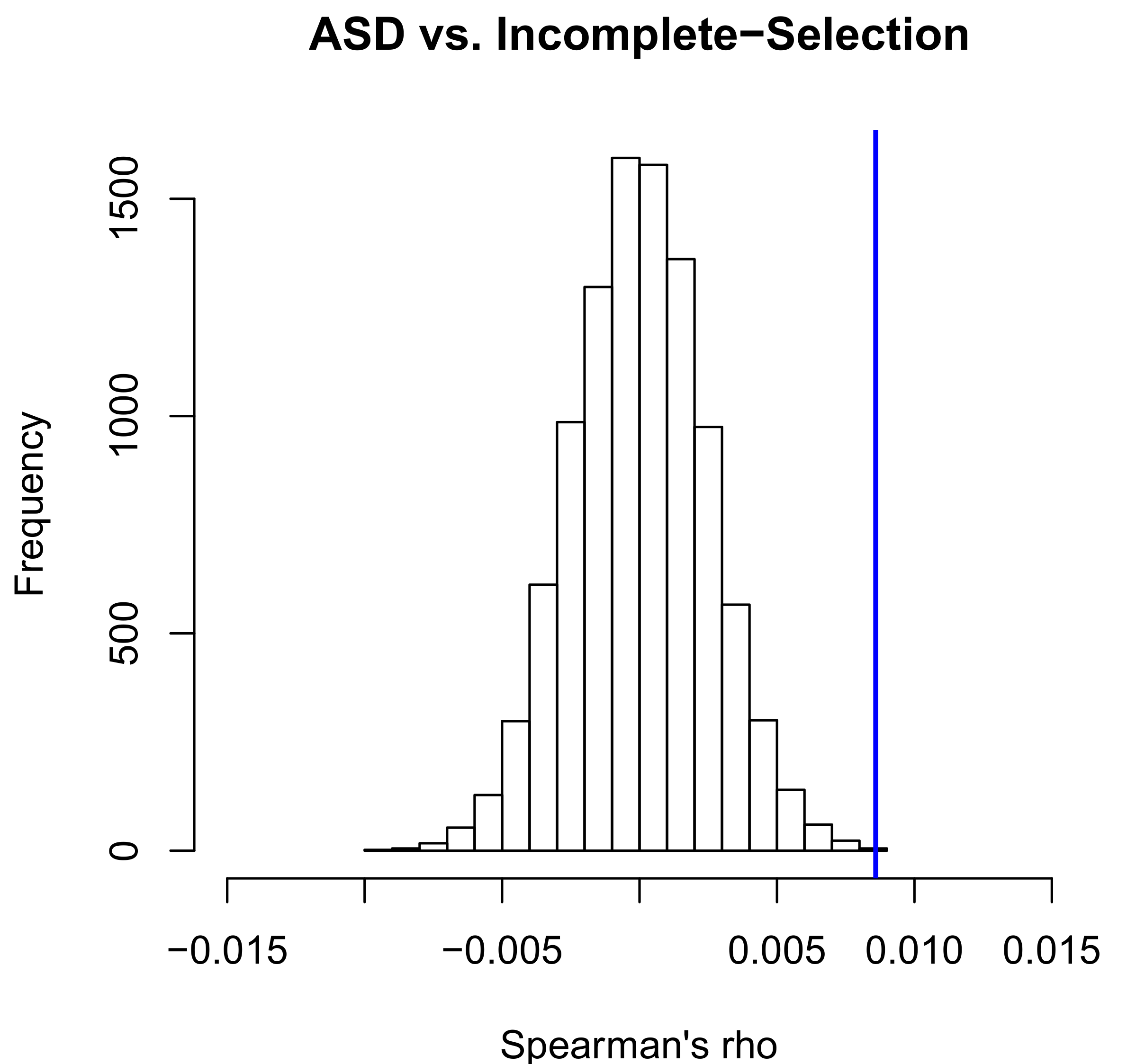
**
