# Supplementary material for: Widespread signatures of positive selection in common risk alleles associated to autism spectrum disorder
Source: PLoS Genet. 2017 Feb 10;13(2):e1006618. doi: 10.1371/journal.pgen.1006618 (PMC5328401; doi:10.1371/journal.pgen.1006618)

**S3 Fig.**: Statistical power of PGC ASD and SCZ GWAS calculated considering different thresholds, a minor allele frequency of 10%, and an effect size of OR = 1.1. The sample size for ASD and SCZ cohorts are those reported in S6 Table.


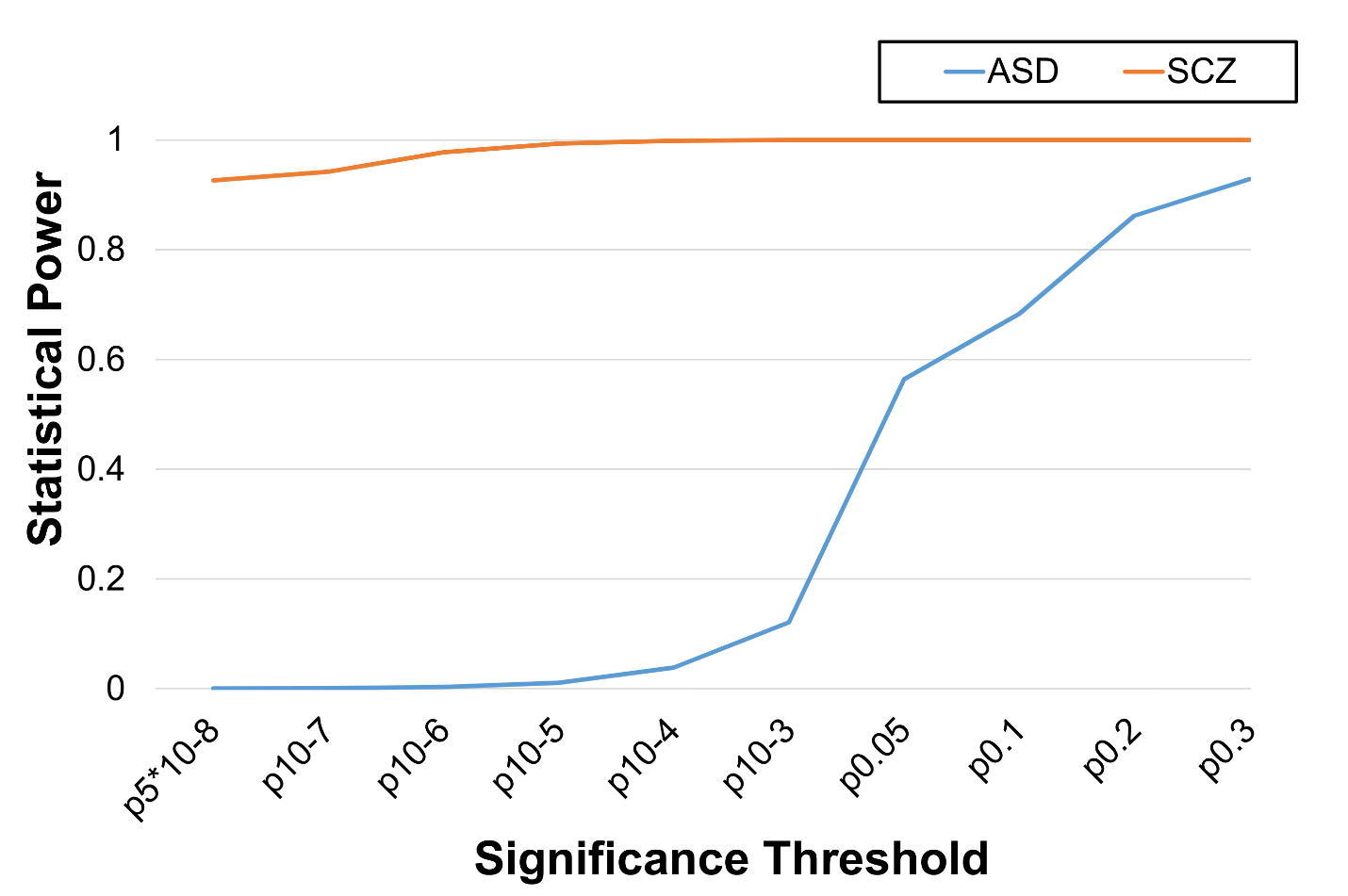

Supplement: S3 Fig — The sample size for ASD and SCZ cohorts are those reported in S6 Table. (DOCX) [file pgen.1006618.s009.docx]
