# Supplementary material for: Widespread signatures of positive selection in common risk alleles associated to autism spectrum disorder
Source: PLoS Genet. 2017 Feb 10;13(2):e1006618. doi: 10.1371/journal.pgen.1006618 (PMC5328401; doi:10.1371/journal.pgen.1006618)

**S4 Fig.**: GO-enrichment results in 100 random permutations. We report the null distribution and the frequency the GOs observed. In the ASD data, we observed enrichment for 53 GOs (p_permutation_<0.01).


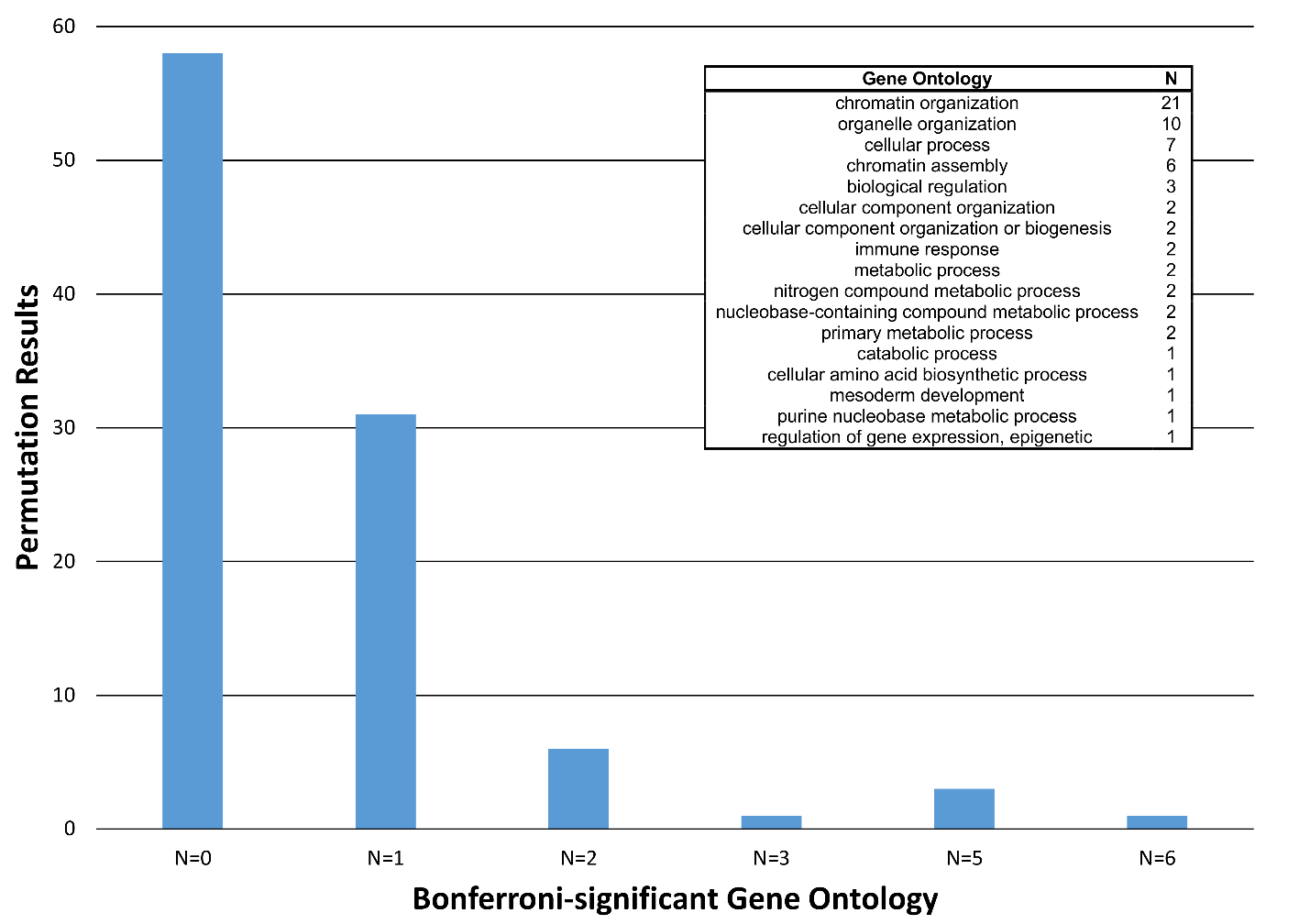

Supplement: S4 Fig — We report the null distribution and the frequency the GOs observed. In the ASD data, we observed enrichment for 53 GOs (ppermutation<0.01). (DOCX) [file pgen.1006618.s010.docx]
